# C-shaped Canal in Second Mandibular Molar: A Case Report

**DOI:** 10.7759/cureus.81784

**Published:** 2025-04-06

**Authors:** Vesela R Vasileva, Tsvetelina Borisova-Papancheva, Denitsa Zaneva-Hristova, Slavena Georgieva, Ana-Maria Miteva

**Affiliations:** 1 Department of Conservative Dentistry and Oral Pathology, Medical University of Varna, Varna, BGR; 2 Department of Conservative Dentistry and Oral Pathology, The Faculty of Dental Medicine, Varna, BGR

**Keywords:** canal configuration, c shape, mandibular second molar, root canal, root canal anatomy

## Abstract

Root canal treatment is a procedure that is often performed in dental practice, both on single-rooted and multi-rooted teeth. Endodontic treatment of multi-rooted teeth can be challenging due to variations in the root canal system. The most common anatomical variation in mandibular second molars is the C-shaped root canal system. In particular, mandibular molars with joined roots, a longitudinal radicular groove on the buccal and/or lingual root surface, and at least one axial cross-section with a "C-shaped" morphology are considered to have C-shaped morphology. To guarantee high-quality care, medication, and C-shaped canal obturation, we need preliminary radiography or cone beam computed tomography (CBCT) to help us determine the diagnosis and best course of action. The purpose of this article is to show а clinical case of a lower second molar with C-shaped anatomy, in which, with magnification, preliminary radiography, and an appropriate system for root canal preparation and obturation, quality treatment is achieved, with the tooth being functional and preserved in the dentition.

## Introduction

Understanding, respecting, and appreciating the anatomy of the root canal, as well as performing cleaning and shaping treatments with care and attention to detail, are essential for successful endodontics. Anatomically, a mandibular molar often resembles a tooth with two roots: one distal and one mesial. The mesial root frequently has two canals that converge in the apical region, while the distal root typically has one [1].

Named for the cross-sectional morphology of the root and root canal, the C-shaped canal was initially described in endodontic literature by Cooke and Cox in 1979 [2]. The pulp chamber of the C-shaped canal has a single ribbon-shaped orifice with a 180° arc (or more) in place of many distinct orifices. In mandibular molars, this orifice begins at the mesiolingual line angle and extends across the buccal region to the pulp chamber's distal face. The root structure may contain a variety of anatomical differences below the orifice level. Those that have a single, ribbon-like, C-shaped canal from orifice to apex and those that have three or more separate canals below the C-shaped orifice are the two primary categories into which these can be divided since single-swath C-shaped canals are the exception rather than the rule [3].

Once identified, the C-shaped canal presents difficulties for obturation and debridement, particularly as it is not always evident if the C-shaped opening on the pulp chamber floor truly extends to the apical third of the root [4]. The presence of a fin or web connecting the separate root canals is the primary anatomical characteristic of C-shaped canals. Conical or square roots with a C-shaped canal are frequently found [5,6]. The following three characteristics must be present in all teeth that qualify as having a C-shaped canal system: fused roots, a longitudinal groove on the lingual or buccal surfaces of the root, and at least one cross-section of the canal belongs to the C1, C2, or C3 configuration [3].

Cooke and Cox [2] stated that it was impossible to diagnose C-shaped canals on the preoperative radiograph, but in the study of Haddad et al. [7], almost all preoperative radiographs showed common characteristics. Overall, radiographic interpretation is more successful when it is based on film combinations rather than individual radiographs ("preoperative and working length radiographs," "preoperative and final radiographs," or "all three radiographs"). Working length radiographs are more useful than preoperative and final ones among the latter, whereas preoperative radiographs are the least useful for diagnosing C-shaped instances [8]. Moreover, C-shaped configurations differ significantly from orifice to apex, making it impossible to accurately anticipate the descending anatomy of the root canal system from the clinical presentation at the orifice level [2,9]. Therefore, the clinical identification and assessment of C-shaped canals may be more predictable with the use of cone beam computed tomography (CBCT) [10].

The aim of our case report is to present a clinical case of a lower second molar with atypical anatomy-C-shaped.

## Case presentation

**Figure 1:**
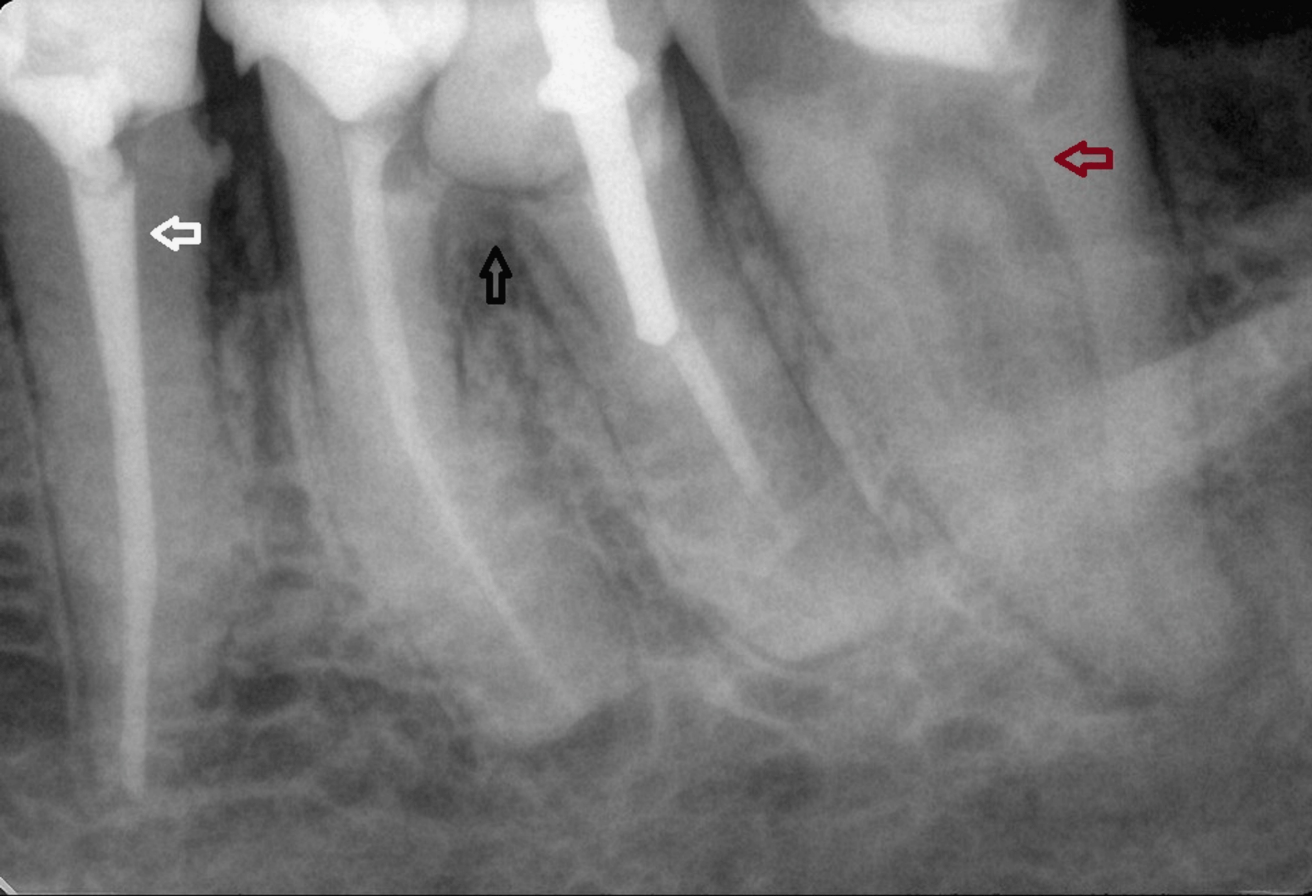
Preoperative radiograph of tooth 37. Tooth 37 with fused roots and the presence of a C-shaped root canal, visible on an intraoral radiograph (red arrow), lesion in the furcation area on tooth 36, with a massive restoration-referred for extraction (black arrow), tooth 35 with satisfactory endodontic treatment (white arrow).

A 45-year-old woman came to the clinic with complaints of constant pain--when eating and when pressing, localized in region 37. Obturation with developed secondary caries was observed during the clinical examination of tooth 37. After a periapical radiographic of tooth 37 and a negative response to the pulp testing, we made a diagnosis of periapical periodontitis with pulp necrosis. The periapical radiograph was taken with a device, Planmeca Romexis (Planmeca Oy, Helsinki, Finland), which revealed an atypical canal configuration with the presence of two root canals that are C-shaped. After evaluating teeth 36 and 35, tooth 36 was selected for extraction (Figure 1).

**Figure 2:**
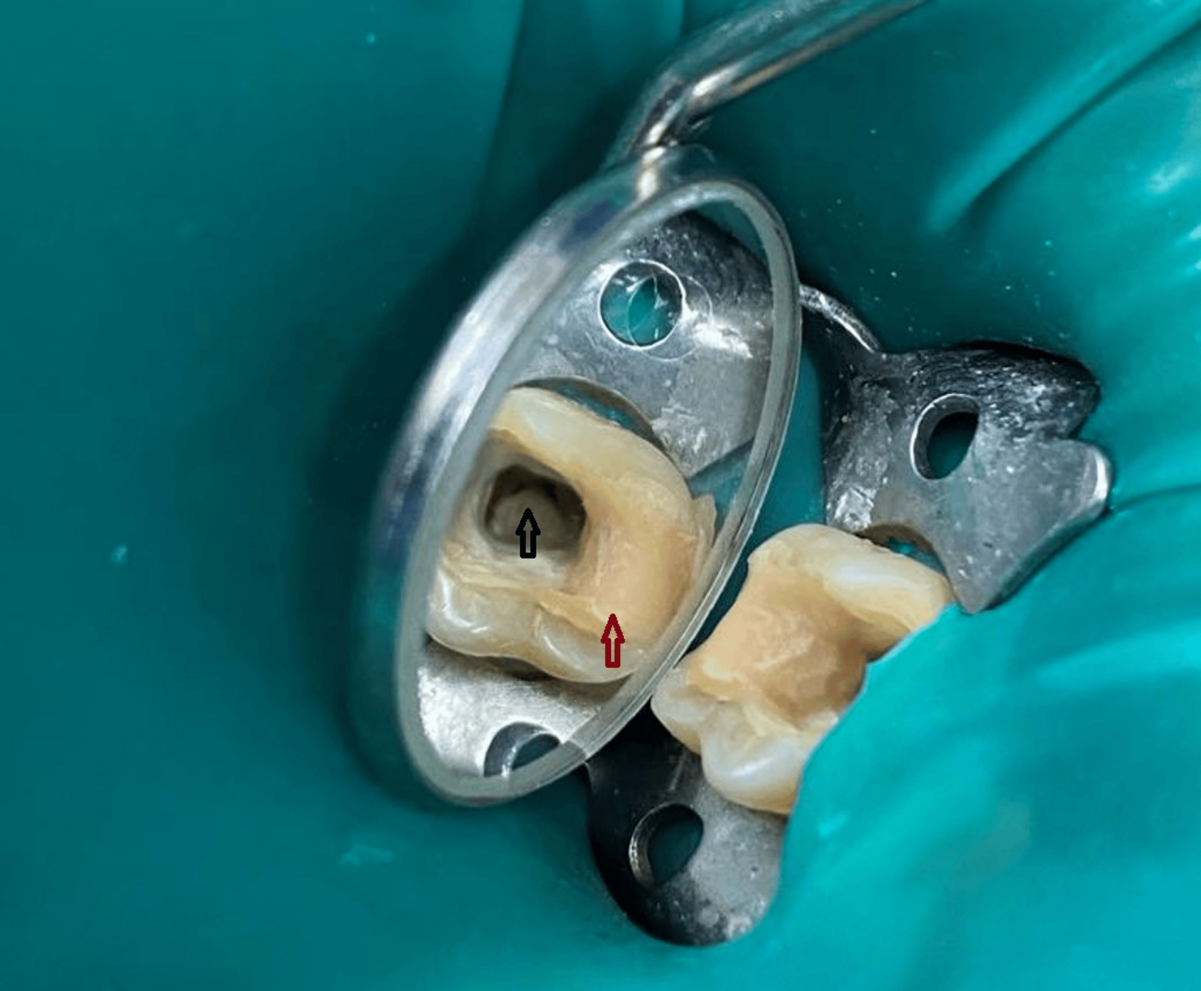
C-shaped canal intraoral. The endocavity with the orifice of the C-shaped canal showed intraoral (black arrow); presence of obturation, which after filling of the root canal was removed and renewed (red arrow)

Following the administration of 1.7 ml 4% articaine hydrochloride Septanest 40 mg/ml 1:200,000 (Septodont, Saint-Maur-des-Fossés, France) local anesthetic, we used a rubber dam to isolate the tooth, cleaned the carious lesion, removed the composite definitive obturation and revealed the pulp chamber. We removed the roof of the pulp chamber with a sterile Endo Z bur (Dentsply Maillefer, Woodbridge, Ontario, Canada) and exposed two wide orifices, mesial and distal, which were C-shaped (Figure 2).

**Figure 3:**
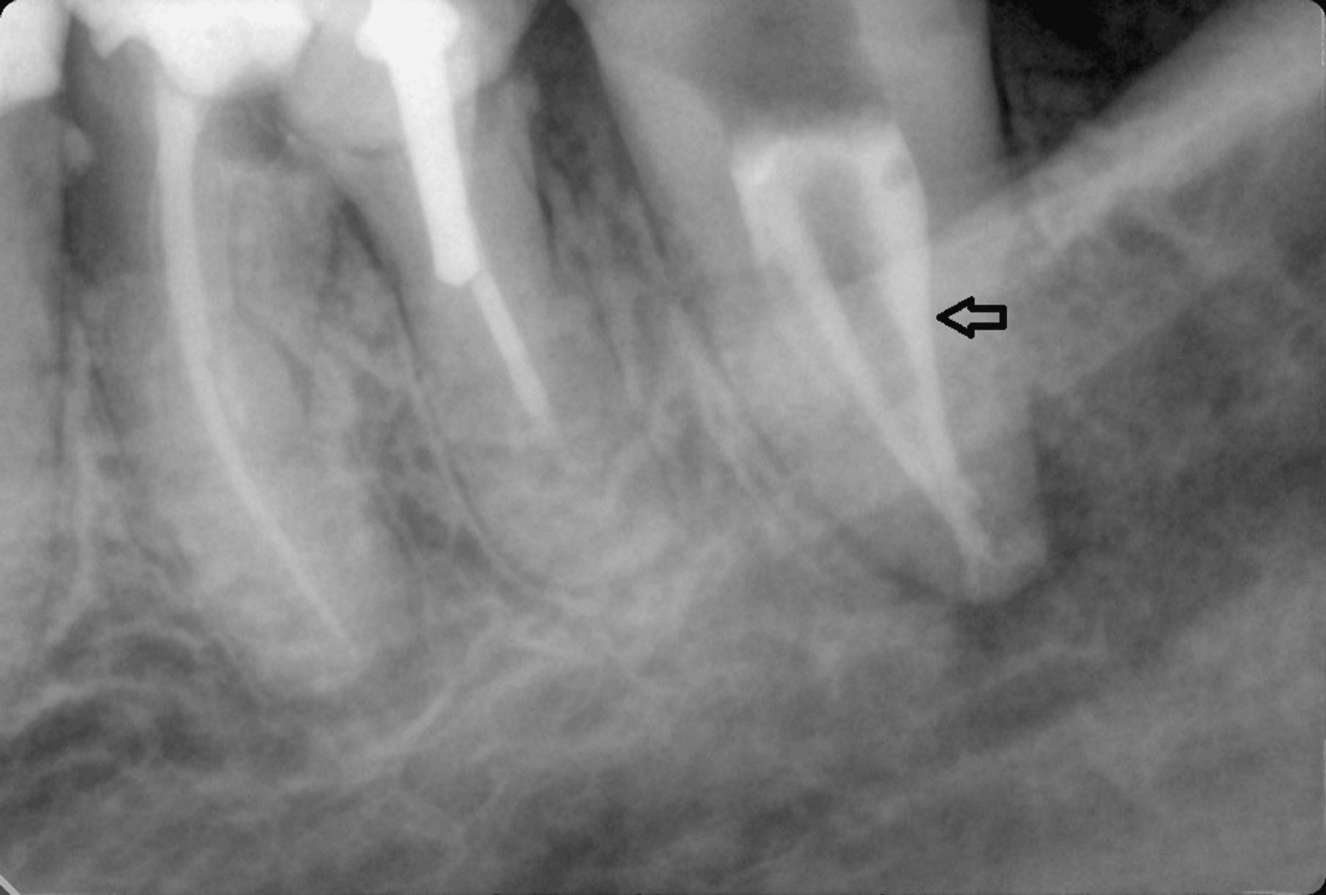
Postoperative radiograph of tooth 37. Obturation of the canals with sealer and gutta percha on tooth 37 (black arrow).

We worked with 3.5X magnification (Univet, Rezzato, Italy) and M320 D Dental Microscope (Leica Microsystems, Wetzlar, Germany). We determined the working length with a 15K file (Dentsply Maillefer) and an apex locator FindPex (Eighteeth, Changzhou, China) -20 mm for the medial canal and 20 mm for the distal one. The machine files we used were from the Wave One Gold system (Dentsply Sirona, Charlotte, NC), and the final one we reached was the Primary file (25/07). Chemical treatment included irrigation with NaOCl 5.25%, saline 0.9%, and citric acid 40%, as well as their sound activation with the EndoActivator (Dentsply Maillefer). After using paper points to dry the root canals, we used Calcipast (Cerkamed, Stalowa Wola, Poland), which resulted in a seven-day temporary obturation with light cure i-PRO LC (iDental) to keep the tooth closed. During the subsequent visit, the patient reported less pain, though it was still present. Examination of the cavity indicated a mild smell coming from the root canals. We applied the same irrigation protocol, including irrigation with Glucochexin 2% (Chlorhexidine 2%, Cerkamed, Stalowa Wola, Poland), due to its high antimicrobial activity against gram-positive and gram-negative bacteria. Тhen we dried and closed the tooth with Calcipast in the canals again for seven days. The patient reported no pain on the subsequent visit, and the tooth had remained asymptomatic for a few days. We filled the root canals using Neo SEALER Flow (Avalon Biomed, Houston, TX) and gutta-percha (for Wave One Gold system, size Primary) using the method of warm vertical condensation. Postoperative radiography showed satisfactory results in filling the C-shaped root canal system (Figure 3).

It shows the presence of a second mesial canal (configuration type 2-1), the treatment and filling of which is due to a meticulous irrigation protocol and activation of the solutions for chemical treatment of the root canals. G-aenial (GC Corporation, Tokyo, Japan) shade A3 composite material was used to reconstruct the tooth. Case follow-up is still pending.

## Discussion

A successful root canal treatment requires extra work since the "C" design is known to show complex canal anatomy; its uneven portions include soft-tissue remnants or diseased debris that may evade complete cleaning or filling processes [11]. The morphology of the pulp chamber floor and the persistence of bleeding or pain when distinct canal orifices are discovered are two specific visible criteria that are used in the clinical detection of C-shaped canals [9].

It could be necessary to adjust the procedure while obturating C-shaped canals. As with regular canals, the mesiolingual and distal canal spaces can be prepared and obturated. However, if lateral condensation is the only technique employed, sealing the buccal isthmus is challenging. The application of thermoplasticized gutta-percha is more suitable since this isthmus might not be prepared with a flare that is sufficient to allow for the deep placement of the spreader [12].

Successful treatment of C-shaped canals requires adequate assessment of the complex root canal morphology. This would be achieved with the use of cone beam computed tomography (CBCT) [13,14]. A major part of eliminating microorganisms is the chemical treatment of root canals. When choosing the right irrigants would lead to the complete elimination of the infected areas in the isthmus. For adequate chemical treatment, we recommend activating the solutions through sonic activation with the EndoActivator.

There are many materials and techniques for filling root canals. It is necessary to evaluate which combination of materials and methods would lead to the hermetic three-dimensional sealing of the root canal system. Bioceramic sealers give good results due to their ability to adapt to the irregularities in the root canal system and thus permanently seal [15]. In this way, they minimize the chance of re-infection.

Our clinical cases emphasize the importance of personalized treatment strategies and the integration of modern technologies to achieve optimal results. Future research should focus on comparative studies evaluating the effectiveness of different techniques and materials for filling complex root canal morphology, such as C-shaped canals, as well as investigating the impact of specific irrigation protocols on long-term prognosis. In our opinion, emphasis should be placed on introducing a standardized protocol for the preparation, irrigation, and filling of teeth with C canals.

## Conclusions

Appropriate radiography, such as CBCT and periapical radiograph, and careful examination of the teeth under magnification are essential for success in such atypical C-shaped root canals. It is crucial to do a clinical evaluation with endodontic explorers and choose a cleaning and shaping strategy suitable for this particular root canal structure.

The correct diagnosis of this condition is essential prior to treatment because the C-shaped canal variant of morphology is uncommon and may cause problems during treatment. Treating a tooth with this configuration presents challenges due to its complex and often unpredictable anatomy, including isthmuses and variations that complicate thorough cleaning and obturation.
